# Exploration of Ear Biometrics Using EfficientNet

**DOI:** 10.1155/2022/3514807

**Published:** 2022-08-31

**Authors:** Aimee Booysens, Serestina Viriri

**Affiliations:** School of Mathematics, Statistics and Computer Science, University of KwaZulu-Natal, Durban, South Africa

## Abstract

Biometrics is the recognition of a human using biometric characteristics for identification, which may be physiological or behavioral. The physiological biometric features are the face, ear, iris, fingerprint, and handprint; behavioral biometrics are signatures, voice, gait pattern, and keystrokes. Numerous systems have been developed to distinguish biometric traits used in multiple applications, such as forensic investigations and security systems. With the current worldwide pandemic, facial identification has failed due to users wearing masks; however, the human ear has proven more suitable as it is visible. Therefore, the main contribution is to present the results of a CNN developed using EfficientNet. This paper presents the performance achieved in this research and shows the efficiency of EfficientNet on ear recognition. The nine variants of EfficientNets were fine-tuned and implemented on multiple publicly available ear datasets. The experiments showed that EfficientNet variant B8 achieved the best accuracy of 98.45%.

## 1. Introduction

**Figure 1:**
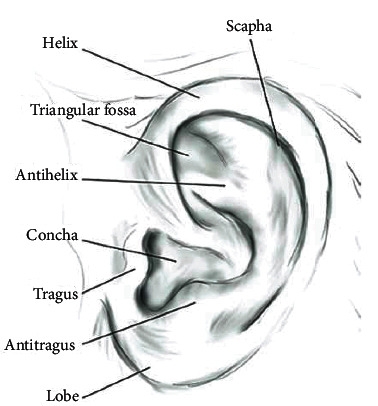
Diagram of the outer ear.

The ear begins to develop in a fetus during the fifth and seventh weeks of pregnancy [1]. At this stage, the face acquires a more distinguishable shape as the mouth, nostrils, and ears begin to form. There is still no exact timeline at which the outer ear is created, but it is accepted that a cluster of embryonic cells connects to establish the ear. These are called auricular hillocks, which begin growing in the lower portion of the neck. The auricular hillocks broaden and intertwine within the seventh week to deliver the ear's shape. Within the ninth week, the hillocks move to the ear canal and are more noticeable as the ear [1]. The external anatomy of the ear can be seen in Figure 1. The growth of the ear in the first four months after birth is linear, and the ear is then stretched in development between the ages of four months and eight years. After this, the ear size and shape are constant until age seventy, increasing in size again.

Biometrics is the recognition of a human using their biometric characteristics, which may be physiological or behavioral. The physiological biometric features are the DNA, face, ear, facial, iris, fingerprint, hand geometry, hand vein, and palm print, and behavioral biometrics are signatures, gait patterns, and keystrokes. Voice is considered as a combination of biometric and physiological characteristics. Numerous systems have been developed to distinguish biometric traits, which have been used in multiple applications, such as forensic investigations and security systems. With the current worldwide pandemic, facial identification has failed due to users wearing masks. However, the human ear has proven more suitable as it is visible. In Table 1, an investigation was done to ascertain the performance, distinctiveness, permanence, collectability, and acceptability of the biometric.

In different physiological biometric qualities, the ear has received much consideration of late as it tends to be said that it is a solid biometric for human acknowledgment [2]. Ear biometric framework is dependable as it does not change and is of uniform tone, and its position is fixed at the center of the face's side. The size of an individual's ear is more critical than a unique finger impression and makes it simpler to capture an image of the subject without necessarily needing to gain information from the subject [2]. There are numerous difficulties in correctly gauging the details of the ear, and these are concealment of the ear by clothing, hair, ear ornaments, and jewelry. Another interference could be the different angles that the image was taken, concealing essential characteristics of the ear's anatomy. These difficulties have made ear recognition a secondary role in identification systems and techniques commonly used for identification and verification.

Although several computer-aided detection models have been developed to identify ears, low accuracy and sensitivity are still significant concerns that misidentify ears. Existing models are also computationally complex and expensive. The contributions of this work are summarized as follows:

1. Implementation of state-of-the-art EfficientNets to develop an effective and inexpensive ear detection system. It is the first time the EfficientNet model is being applied to classify ears.
2. The proposed model accuracy through EfficientNet.
3. Finally, benchmark datasets were used to evaluate the performance of the model.

The remainder of the work is structured as follows: Section 2 presents related works, and Section 3 presents detailed data and methodology explored in this study. The experimental results and discussion are provided in Section 4, and Section 5 concludes the paper.

## 2. Related Work

This section presents different algorithms using the convolutional neural network (CNN) for ear identifications, and a summary of the related works is shown in Table 2.

Emeršič et al. [3] organized the dataset of the UERC which was used for the benchmark, training, and testing sets. In the completion, it was seen that handcrafted feature extraction methods, such as LBP [13] and patterns of oriented edge magnitudes (POEM) [14], and CNN-based feature extraction methods were used to obtain the ear identification. The challenges were to find methods to remove occlusions such as earrings, hair, other obstacles, and background from the ear image. The occlusion was done by creating a binary ear mask, and then the system recognition was done using the handcrafted features. Another proposed approach was to calculate the score of matrices from the CNN-based features and handcrafted features when they are fused, and a 30% detection rate was achieved.

Tian and Mu [4] applied a CNN to ear recognition in which they designed a CNN—it was made up of three convolutional layers, a fully connected layer, and a softmax classifier. The database used was USTB ear, which consisted of 79 subjects with various pose angles. The images utilized excluded earrings, headsets, or similar occlusions. Chowdhury et al. [15] proposed an ear biometric recognition system that uses local features of the ear and then uses a neural network to identify the ear. The method estimates where the ear could be in the input image and then takes the edge features from the identified ear. After identifying the ear, a neural network matches the extracted feature with a feature database. The databases used in this system were AMI, WPUT, IITD, and UERC, which achieved an accuracy of 70.58%, 67.01%, 81.98%, and 57.75%, respectively.

Raveane et al. [5] presented that it is difficult to precisely detect and locate an ear within an image, this challenge increases when working with the variable condition, and this could also be because of the odd shape of the human ears as well as lighting conditions and the changing profile shape of an ear when photographed [5]. The ear detection system used multiple CNNs, combined with a detection grouping algorithm, to identify an ear's presence and location. The proposed method matches other methods' performance when analyzed against clean and purpose-shot photographs, reaching an accuracy of upward of 98%. It outperforms them with a rate of over 86% when the system is subjected to non-cooperative natural images where the subject appears in challenging orientations and photographic conditions.

Multiple scale faster region-based convolutional neural network (Faster R-CNN) to detect ears from 2D profile images was proposed by Zhang and Mu [6]. This method was used by taking three regions of different scales that are detected to defer the information from the ear location within the context of the ear in the image, which was done to extract the ear correctly. The system was tested with 200 web images that achieved a 98% accuracy. Other experiments conducted were on the Collection J2 of the University of Notre Dame Biometrics Database (UND-J2) and University of Beira Interior Ear dataset (UBEAR); these achieved a detection rate of 100% and 98.22%, respectively, but these datasets contained large occlusions, scale, and pose variation.

Kohlakala and Coetzer [7] presented semi-automated and fully automated ear-based biometric verification systems. CNN and morphological postprocessing manually identify the ear region. It is used to classify ears in either the foreground or background of the image. The binary contour image applied the matching for feature extraction, and this was done by implementing a Euclidean distance measure, which had a ranking to verify for authentication. The Mathematical Analysis of Images Ear database and the Indian Institute of Technology Delhi Ear database were two databases, which achieved 99.20% and 96.06%, respectively.

Geometric deep learning (GDL) generalizes CNNs to non-Euclidean domains, presented by [8] Tomczyk and Szczepaniak. It used the convolutional filters with a mixture of Gaussian models. These filters were used so that the images could be easily rotated without interpolation. It shows the published experimental results that the approach did the rotational equivalence property to detect rotated structures. Still, it does not need labor-intensive training on all rotated and nonrotated images.

Alshazly et al. [9] presented and compared ear recognition models built with handcrafted and CNN features. The paper took seven performing handcrafted descriptors to extract the discriminating ear image. They then took the extracted ear and trained it using support vector machine (SVM) to learn a suitable model. They then used CNN-based models, which used a variant of the AlexNet architecture. The results obtained on three ear datasets showed the CNN-based models' performance increased by 22%. This paper also investigated if the left and right ears have symmetry. The results obtained by the two datasets indicate a high impact of balance between the ears.

Alkababji and Mohammed [10] presented the use of a deep learning item detector called faster region-based convolutional neural network (Faster R-CNN) for ear detection. This CNN is used for feature extraction. It used the principal component analysis (PCA) and a genetic algorithm for feature reduction and selection. It also used a connected artificial neural network as the matcher. The results achieved an accuracy of 97.8% success rate.

Jamil et al. [11] build and train a CNN model for ear biometrics in various uniform illuminations measured using lumens. They considered that their work was the first to test the performance of CNN on underexposed or overexposed images. The results showed that for images with uniform illumination with a luminance of above 25 lux achieved a result of 100%. The CNN model had problems recognizing images when the lux was below ten, but produced an accuracy of 97%. This result shows that CNN architecture performs just as well as the other systems. It was found that the dataset had rotations which affected the results.

Hansley et al. [12] presented an unconstrained ear recognition framework that was better than the current state-of-the-art systems using publicly available databases. They developed CNN-based solutions for ear normalization and description. This was done using handcrafted descriptors, which were fused to improve recognition. This was done in two stages. The first stage was to find the landmark detectors, which were untrained scenarios. The next step was to generate a geometric image normalization to boost the performance. It was seen that the CNN descriptor was better than other CNN-based works in the literature. The obtained results were higher than different reported results for the UERC challenge.

## 3. Data and Methods

### 3.1. Dataset

In this study, all the experiments were performed with numerous public ear datasets; an explanation of these datasets is provided below. UBEAR, EarVN1.0, IIT, ITWE, and AWE databases are best suited for ear identification due to their large data size. However, it shows that EarVN1.0 has the foremost prominent usage during age estimation using CNN techniques. It is an appropriate dataset for ear images taken in a controlled environment, while ITWE is compatible for classifying ears in an uncontrolled environment, a summary of the datasets is shown in Table 3.

#### 3.1.1. Mathematical Analysis of Images (AMI) Ear Database

The AMI Ear database [19] was collected at the University of Las Palmas. The database comprises 700 ear images of 100 distinct Caucasian male and female adults between the ages of 19 and 65. All images within the database were taken under an equivalent illumination and a glued camera position. Both the left- and right-hand sides of the ears were captured. The pictures obtained were cropped to form the ear area covering almost half the image. The pose of the images varies in yaw and servery in pitch angles, and this dataset is often found publicly.

#### 3.1.2. The Indian Institute of Technology (IIT) Delhi Ear Database

The IIT database [16] was collected by the Indian Institute of Technology Delhi in New Delhi between October 2006 and June 2007. The database is formed from 421 images of 121 distinct adults of both male and female. All images were taken inside the environment, with no significant occlusions present, and only the right-hand side of the ear was captured. The pictures obtained in the dataset were both raw and normalized. The normalized images were in grayscale and of size 272 × 204 pixels.

#### 3.1.3. The University of Beira Ear (UBEAR) Database

The University of Beira presented the UBEAR database [25]. The database comprises 4429 images of 126 subjects, and these were of both males and females. The images were taken under varying lighting conditions, and angles and partial occlusions were present. These images were of both the left- and right-hand sides of the ear.

#### 3.1.4. The Annotated Web Ear (AWE) Database

The AWE database [18] is a set of public figures from web images. The database was formed from 1000 images of 100 different subjects whose sizes vary and were tightly cropped. Both the left- and right-hand sides of the ears were taken.

#### 3.1.5. EarVN1.0

The EarVN1.0 database [22] comprises 28412 images of 164 Asian male and female subjects, and left- and right-hand sides of the ear were captured. Collection was during 2018 and is formed from unconstrained conditions, including camera systems and lighting conditions. The pictures are cropped from facial images to obtain the ears, and the pictures have significant variations in pose, scale, and illumination.

#### 3.1.6. The Western Pomeranian University of Technology Ear (WPUTE) Database

The Western Pomeranian University of Technology Ear (WPUTE) database [20] was obtained within the year 2010 to gauge the ear recognition performance for images obtained within the wild. The database contains 2071 ear images belonging to 501 subjects. The images were of various sizes and were of both the left- and right-hand sides of the ear, and these were taken under different indoor lighting conditions and rotations. There were some occlusions included in the database, and these were the headset, earrings, and hearing aids.

#### 3.1.7. The Unconstrained Ear Recognition Challenge (UERC)

The Unconstrained Ear Recognition Challenge (UERC) database [21] was obtained in 2017, then extended in 2019, and is a mix of two databases that currently exist and a newly created one. The database contains 3706 subjects with 11804 ear images, and the database ears have both right- and left-hand side images.

#### 3.1.8. In-the-Wild Ear (ITWE) Database

The In-the-Wild Ear (ITWE) database [23] was created for recognition evaluation and has 2058 total images, and 231 male and female subjects. A boundary box obtained these images of the ear, and coordinates of those boundary boxes were released with the gathering. The pictures contained cluttering backgrounds and were of variable size and determination. The database includes both left- and right-hand sides of the ear, but no differentiation was given about the ears.

#### 3.1.9. The University of Science and Technology Beijing (USTB) Ear Database

The University of Science and Technology Beijing (USTB) Ear database [17] contained cropped ear and head profile images of male and female subjects split into four sets. Dataset one includes 60 subjects and has 180 images of right close-up ears during 2002. These images were taken under different lightings and experienced some shearing and rotation. Dataset two contains 77 subjects, has 308 images of the right-hand side ear approximately 2 meters away from the ear, and these images were taken in 2004. These images were taken under different lighting conditions. Dataset three contains 103 subjects and has 1600 images, and these images were taken during the year 2004. The images are on the proper and left rotation, and therefore, the images are of the dimensions 768 × 576 pixels. The dataset contains 25500 images of 500 subjects; these were obtained from 2007 to 2008; the subject was in the center of the camera circle. The images were taken when the subject looked upward, downward, and at eye level. The images during this dataset contained different yaw and pitch poses. The databases are available on request and accessible for research.

#### 3.1.10. The Carreira-Perpinan (CP) Ear Database

The Carreira-Perpinan (CP) [24] Ear database is an early dataset of the ear utilized for ear recognition systems. It was created in 1995 and contained 102 images with 17 subjects. The images were captured in a controlled environment, and therefore, the images include variability in minor pose variation.

#### 3.1.11. The Indian Institute of Technology Kanpur (IITK) Ear Database

The Indian Institute of Technology Kanpur (IITK) is an ear database [26] that the Institute of Technology of Kanpur compiled. The database is split into three sets, and the first set consists of 190 male and female subjects of profile images. The total number of images was 801. The second dataset also contained 801, and with a total of 89 subjects, these images had variations in pitch angle. The third dataset contains 1070 images of an equivalent of 89 subjects, but with a variation in yaw and angle.

#### 3.1.12. The Forensic Ear Identification Database (FEARID)

The Forensic Ear Identification Database (FEARID) [27] is different from other databases as it only includes the ear prints. These contain no occlusions, variable angles, or illumination. Though there is no mention of any variables, other influences like the force the ear was pressed against the scanner and the scanner's cleanliness need to be considered. This database comprised 7364 images of 1229 subjects. This database was used for forensic application and not for biometric use.

#### 3.1.13. The University of Notre Dame (UND) Database

The University of Notre Dame (UND) database contains [28] many subsets of 2D and 3D ear images. These images were appropriated over a period from 2003 to 2005. The database contains 3480 3D images from 952 male and female subjects and 464 2D images from 114 male and female subjects. These images were taken in different lighting conditions, yaw, pitch poses, and angles. The images are only of the left-hand side of the ear.

#### 3.1.14. The Face Recognition Technology (FERET) Database

The Face Recognition Technology (FERET) database [29] is a sizeable facial image database and was obtained between the years 1995 to 1996. It contains 1564 subjects and has a total of 14126 images. These images were collected for face recognition and were of the left- and right-hand profile images, which made them perfect for 2D ear recognition.

#### 3.1.15. The Pose, Illumination and Expression (PIE)

Carnegie Mellon University obtained the Pose, Illumination and Expression database [30], which contains 40000 images and 68 subjects. The images are of the facial profile and have different poses, illuminations, and expressions.

#### 3.1.16. The XM2VTS Ear Database

The XM2VTS Ear database [31] is frontal and profile facial images from the University of Surrey; the database contains 295 subjects and 2360 images captured during controlled conditions. These images were a set of cropped images of 720 × 576 pixel size and were from video data.

#### 3.1.17. The West Virginia University (WVU) Ear Database

The West Virginia University (WVU) Ear database [32] is a video database and is formed from 137 subjects. The system was an advanced capturing procedure that allowed them to capture the ear at different angles; these images included earrings and eyeglasses.

### 3.2. Preprocessing

**Figure 2:**
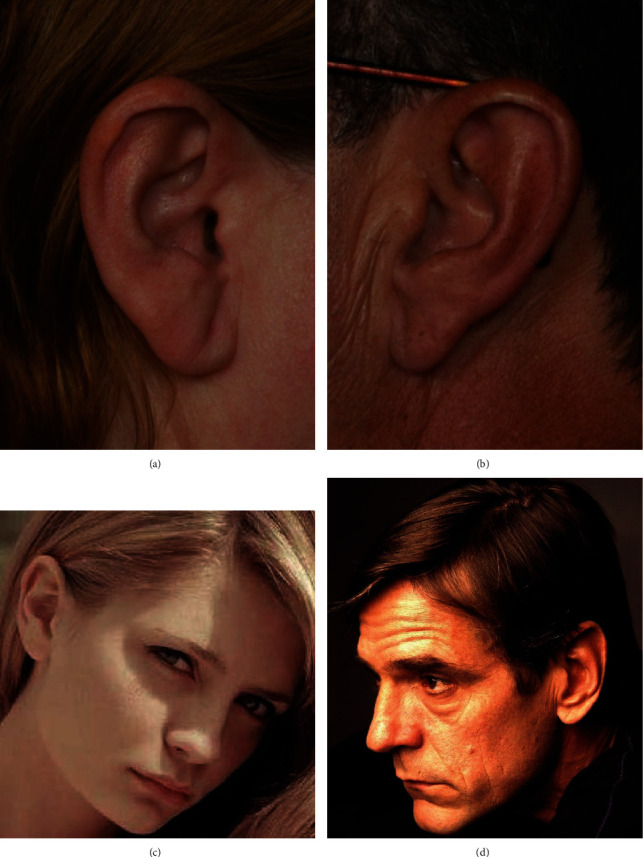
Examples of original ear images. (a) Example of a 2D profile image for a female. (b) Example of a 2D profile image for a male. (c) Example of a facial image for a female. (d) Example of a facial image for a male.

**Figure 3:**
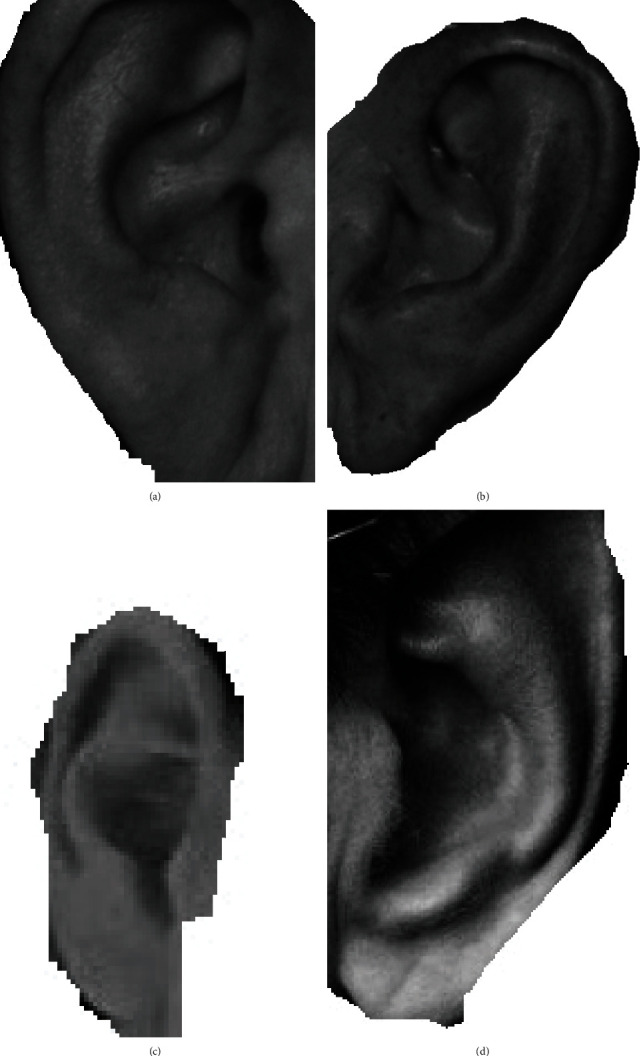
Examples of extracted ear images. (a) Example of ear extracted from 2D profile image for a female. (b) Example of ear extracted from 2D profile image for a male. (c) Example of ear extracted from facial image for a female. (d) Example of ear extracted from facial image for a male.

Image preprocessing is a considerable part of the deep learning task. Most CNN models generally require a large dataset to learn to discriminate features suitably for making predictions and obtaining a good performance. As images in the datasets are of different sizes, the inputted images need to be resized to conform to all the other CNN models, but the features need to be preserved when resizing is performed. The examples of the original and the preprocessed images are shown in Figures 2 and 3.

### 3.3. Transfer Learning

In this study, the concept of transfer learning was adopted and helped with the pretrained CNN model for large datasets to learn features of the target (right and left ears). It will transfer the features learned by the deep CNN models on other CNN models to this dataset. The number of deep CNN model parameters increases as the network gets deeper, which is used to achieve improved efficiency.

**Figure 4:**
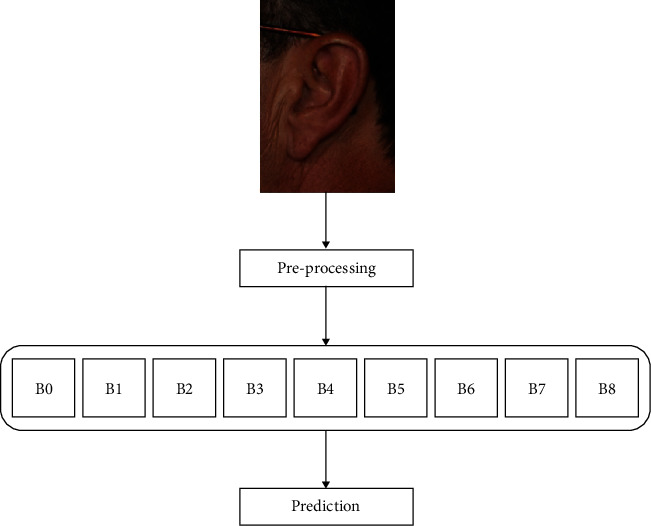
Block structure of the proposed model.

Hence, it requires many datasets for training, making it computationally complex and applying these models directly on small and new dataset results in feature extraction bias, overfitting, and poor generalization. The pretrained CNN modified and fine-tuned its structure to suit the dataset given. This concept of transfer learning is computationally expensive, has less training time, overcomes limitations of the dataset, improves performance, and is faster than training a model from the beginning. The pretraining CNN model fine-tuned in this work is the EfficientNets. The proposed structure is represented in Figure 4.

### 3.4. EfficientNet Architecture

EfficientNet is a lightweight model based on the auto machine learning framework to develop a baseline EfficientNet B0 network and uniformly scaled up the depth, width, and resolution using a simplified and effective compound coefficient to improve EfficientNet models B1–B8. The models performed efficiently and attained superiority over the existing CNN models on the other CNN datasets. EfficientNets are smaller and only require a few parameters, and they are faster and more generalizable to obtain higher accuracy on other datasets' poplar for the transfer learning task. The proposed study fine-tuned EfficientNet models B0–B8 on the dataset to detect the ears. In transferring the pretrained EfficientNets to the ear dataset, the models were fine-tuned by adding a global average pooling to reduce the number of parameters and fix overfitting. The dense layers follow the global average pooling with a ReLU activation function and a dropout rate of 0.4 before the output last layer [33]. This is done with the softmax activation function to determine the probabilities of the input data to represent the ears, and this can be seen in

(1) $$\begin{array}{c} \sigma {\left(q\right)}_{i}=\frac{e^{q_{i}}}{\sum_{N}^{y=1}e^{q_{y}}}\text{,} \end{array}$$

where *σ* is the softmax activation function, *q* represents the input vector to the output layer, *i* is depicted from the exponential element *e*^*q*_*i*_^, *N* is the number of classes, and *e*^*q*_*y*_^ represents the output vector of the exponential function.

It is known that many iterations could lead to model overfitting, while too few can cause model underfitting; this study used an early stopping strategy. It configured approximately 90 training iterations before terminating, this was to cater for early stopping to improve performance, and this was applied to control overfitting and used gradient descent. The EfficientNet B0-B8 models were trained with 100 iterations (epochs). The batch size for each iteration was 32, and the momentum equals 0.2 and was regulated. At the same time, categorical cross-entropy is the loss function used to update weights at each iteration. Hyperparameters used were evaluated and found to perform optimally, and this can be defined in

(2) $$\begin{array}{c} \alpha =\alpha -n\cdot {△}_{\alpha }J\left(\alpha ;x^{i};y^{i}\right)\text{,} \end{array}$$

where △_*α*_*J* is the gradient of the loss with regard to *α*, *n* is the defined learning rate, *α* is the weight vector, while *x* and *y* are the respective training sample and label.

## 4. Results and Discussion

Various EfficientNet variants were fine-tuned on all the ear datasets to detect the ear. Each dataset is split into 20% training and 80% test sets. The experiments were entirely performed using Keras deep learning framework using the TensorFlow backend. The models were evaluated using the popular evaluation metrics, equation (3)–(7) (accuracy, sensitivity, specificity, and area under the curve). The performances of all experiments are evaluated by using a series of confusion matrix-based performance metrics.

The confusion matrices are used to evaluate the classifiers, with true positives (TPs) representing the ears that are correctly classified as positive, true negatives (TNs) representing the ears that are correctly classified as negative, false positives (FPs) representing the ears that are incorrectly classified as positive, and false negatives (FNs) representing the ears being incorrectly classified as negative.

### 4.1. Specificity

It is the ratio of correctly classified negative instances by a model to the overall number of true-negative instances being tested, equation (5).

### 4.2. Accuracy

It is a measure that indicates the ratio of all the correctly recognized cases to the overall number of cases. While this metric generally gives a decent reflection of the classifier, it may not reflect a classifier's true performance in a scenario where there is an uneven class distribution. Accuracy can be computed using the following formula, equation (3).

### 4.3. Sensitivity

It is the ratio of all correctly classified positive instances by a model to the overall number of positive classifications by a model. A low precision indicates that a model suffers from high false positives. Precision can be computed using the following formula, equation (4).

(3) $$\begin{array}{c} accuracy=\frac{TP+TN}{TP+FP+TN+FN^{\prime }}, \end{array}$$

(4) $$\begin{array}{c} sensitivity=\frac{TP}{TP+FN^{\prime }}, \end{array}$$

(5) $$\begin{array}{c} specificity=\frac{TN}{TN+FP^{\prime }}, \end{array}$$

(6) $$\begin{array}{c} TPR=sensitivity \end{array}$$

(7) $$\begin{array}{c} FPR=1-sensitivity \end{array}$$

**Figure 5:**
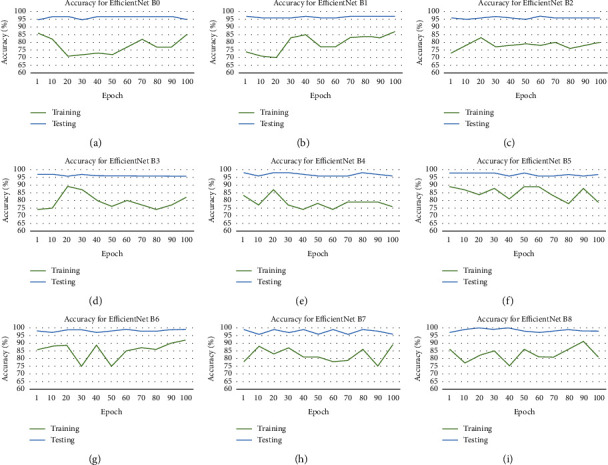
Accuracy for the ear dataset of each EfficientNet. (a) Accuracy for EfficientNet B0. (b) Accuracy for EfficientNet B1. (c) Accuracy for EfficientNet B2. (d) Accuracy for EfficientNet B3. (e) Accuracy for EfficientNet B4. (f) Accuracy for EfficientNet B5. (g) Accuracy for EfficientNet B6. (h) Accuracy for EfficientNet B7. (i) Accuracy for EfficientNet B8.

**Figure 6:**
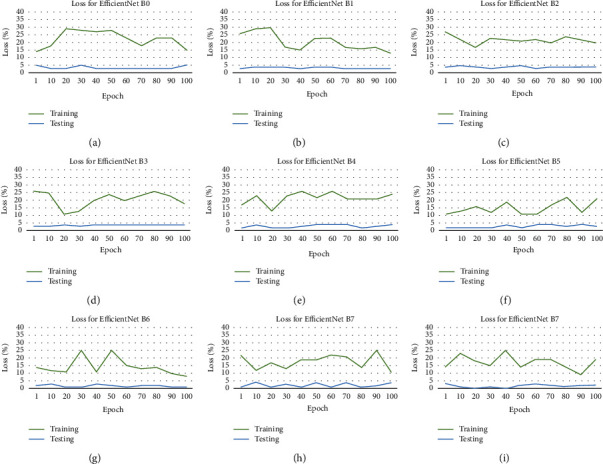
Loss for the ear dataset of each EfficientNet. (a) Loss for EfficientNet B0. (b) Loss for EfficientNet B1. (c) Loss for EfficientNet B2. (d) Loss for EfficientNet B3. (e) Loss for EfficientNet B4. (f) Loss for EfficientNet B5. (g) Loss for EfficientNet B6. (h) Loss for EfficientNet B7. (i) Loss for EfficientNet B8.

The results obtained are presented in Figures 5 and 6 this is the accuracy and loss of these datasets. The various EfficientNet models average at the 100 epochs, and the accuracy is determined using the test set. The models performed at extracting and learning discriminative features from the dataset. EfficientNet B8 attains the best accuracy 98.45%, and the EfficientNet results are noted in Table 4.

**Figure 7:**
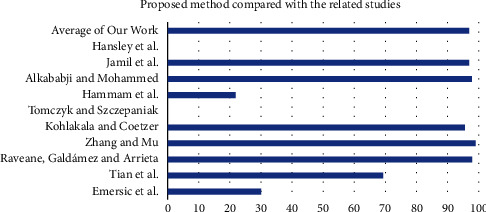
Proposed method compared with the related studies.

An advantage of EfficientNets is that they are smaller with fewer parameters and faster, and obtain transfer learning successfully from the datasets. The worst performing EfficientNet is B2, as shown in Table 4. Even though it has minimal parameters, the reason that this performed poorly could have been because the images were down-sampled. This was done to conform to the model's image input size. It can be seen that performance improves as the model gets deeper. EfficientNet B0 started poorly, beginning to converge from the 30 iteration, with little noise, until the 30 iteration and then stabilized until 50 iteration, when overfitting started. The best performing EfficientNet is B8, as shown in Table 4, and this is because of the large number of parameters. It began to converge from the 60 iteration and then stabilized until 90 iteration, when overfitting started. It is found that when the dataset is a large and equal number of classes, the results achieved were high. Determining the most suitable hyperparameters was one of the challenges faced and the overfitting, which was limited due to the data samples. The results of the proposed methods compared with related studies are presented in Figure 7.

## 5. Conclusion

This study investigated and implemented EfficientNet models to automatically identify ears on the most prominent and publicly available datasets. EfficientNets that achieved state-of-the-art performance over other architectures to maximize accuracy and efficiency were explored and fine-tuned on profile images. The fine-tuning technique is valuable to utilize rich generic features learned from significant dataset sources such as ImageNet to compliment the lack of annotated datasets affecting ear domains. The experimental results show the effectiveness of EfficientNets in extracting and learning distinctive features from the ear images and then classifying them into a left or right suitable class. Out of the nine EfficientNet variants explored in this study, the EfficientNet B8 outperformed the others, as evident in Table 5 and depicted in Figure 7. One of the significant downfalls of the proposed approach is training the model on small datasets and training on images with low resolutions. These limitations can easily result in significant overfitting. To overcome this, you need to have compelling image preprocessing techniques. Although the proposed methodology is specified to do ear detection, it could be extended to detect other parts of the face, given the right set of datasets.

## Figures and Tables

**Table 1:** Summary of biometric characteristics.

Biometric identifier	Biometric type	Distinctiveness	Permanence	Collectability	Performance	Acceptability
DNA	Physiological	High	High	Low	High	Low
Ear	Physiological	Medium	High	Medium	Medium	High
Face	Physiological	Low	Medium	High	Low	High
Facial	Physiological	High	Low	High	Medium	High
Fingerprint	Physiological	High	High	Medium	High	Medium
Gait	Behavioral	Low	Low	High	Low	High
Hand geometry	Physiological	Medium	Medium	High	Medium	Medium
Hand vein	Physiological	Medium	Medium	Medium	Medium	Medium
Iris	Physiological	High	High	Medium	High	Low
Keystroke	Behavioral	Low	Low	Medium	Low	Medium
Odor	Physiological	High	High	Low	Low	Medium
Palm print	Physiological	High	High	Medium	High	Medium
Retina	Physiological	High	Medium	Low	High	Low
Signature	Behavioral	Low	Low	High	Low	High
Voice	Combination of physiological and behavioral	Low	Low	Medium	Low	High

**Table 2:** Summary of the related works.

Author	Dataset	Accuracy	Summary
Emeršič et al. [3]	NA	30	It was a handcrafted feature extraction method, such as LBP and patterns of oriented edge magnitudes (POEM), and CNN-based feature extraction methods were used to obtain the ear identification

Tian and Mu [4]	AMI, WPUT, IITD, and UERC	70.58, 67.01, 81.98, and 57.75	This system used deep convolutional neural network (CNN) to ear recognition. There were occlusions like no earrings, headsets, or similar occlusions

Raveane et al. [5]	NA	98	This system used variable conditions, and this could also be because of the odd shape of the human ears and changing lighting conditions

Zhang and Mu [6]	Notre Dame Biometrics database and University of Beira Interior Ear dataset	100 and 98.22	This system contained large occlusions, scale, and pose variation

Kohlakala and Coetzer [7]	Mathematical Analysis of Images Ear database and Indian Institute of Technology Delhi Ear database	99.2 and 96.06	It is used to classify ears in either the foreground or background of the image. The binary contour image applied the matching for feature extraction, and this was done by implementing a Euclidean distance measure, which had a ranking to verify for authentication

Tomczyk and Szczepaniak [8]	NA	NA	It shows the published experimental results that the approach did the rotation equivalence property to detect rotated structures

Hammam et al. [9]	Three ear datasets but not stated	22	The paper took seven performing handcrafted descriptors to extract the discriminating ear image. They then took the extracted ear and trained it using support vector machines (SVM) to learn a suitable model

Alkababji and Mohammed [10]	NA	97.8	It used the principal component analysis (PCA) and a genetic algorithm for feature reduction and selection

Jamil et al. [11]	Very underexposed or overexposed database	97	They considered that their work was the first to test the performance of CNN on very underexposed or overexposed images

Hansley et al. [12]	UERC challenge	NA	This was done using handcrafted descriptors, which were fused to improve recognition

**Table 3:** Summary of datasets.

	Database	Year	Number of subjects	Number of images	Left ear count	Right ear count	Total ears	Image size	Country	Sides
1	Institute of Technology Delhi Ear Database (IIT Delhi-I) [16]	2007	121	471		471	471	272 × 204	India	Right
	Institute of Technology Delhi Ear Database (IIT Delhi-II) [16]	NA	221	793		793	793	272 × 204	India	Right

2	The University of Science and Technology Beijing (USTB ear I) [17]	2002	60	185		185	185	Varied	China	Right
	The University of Science and Technology Beijing (USTB ear II) [17]	2004	77	308		308	308	Varied	China	Right

3	The Annotated Web Ears database (AWE) [18]	2016	100	1000	500	500	1000	Varied	Slovenia	Both
	The Annotated Web Ears database extended (AWE extend) [18]	2017	346	4104	2052	2052	4104	Varied	Slovenia	Both

4	Mathematical Analysis of Images Ear database (AMI) [19]	NA	106	700	420	280	700	492 × 702	Spain	Both

5	The West Pomeranian University of Technology Ear database (WPUTE) [20]	2010	501	2071	829	1242	2071	Varied	Poland	Both

6	Unconstrained Ear Recognition Challenge database (UERC) [21]	2017	3706	11804	5902	5902	11804	Varied	Slovenia	Both

7	EarVN1.0 [22]	2018	164	28412	14206	14206	28412	Varied and low resolution	Vietnam	Both

8	The In-the-Wild Ear database (ITWE) [23]	2015	55	605	424	181	605	Varied	Slovenia	Both

9	The Carreira-Perpinan (CP) [24]	1995	17	102	102		102	Varied	NA	Left

10	The University of Beira Ear Database (UBEAR) [25]	2011	126	4430	2215	2215	4430	1280 × 960	Mozambique	Both

11	Indian Institute of Technology Kanpur (IITK) [26]	2011	801	190	95	95	190	Varied	India	Both
12	The Forensic Ear Identification Database (FEARID) [27]	2005	1229	1229	615	614	1229	Varied	UK, Italy, and Netherlands	Both

13	University of Notre Dame (UND) [28]	2006	3480	952	952		952	Varied	France	Left

14	The Face Recognition Technology database $FERET) [29]	2010	9427	4745	3796	949	4745	Varied	Spain	Both

15	The Pose, Illumination and Expression (PIE) [30]	2002	40000	68	34	34	68	Varied	USA	Both

16	The XM2VTS Ear database [31]	NA	2360	295	89	206	295	720 × 576	UK	Both

17	The West Virginia University (WVU) [32]	2006	460	402	402		402	Varied	USA	Left

**Table 4:** Performance of EfficientNet models.

Epoch	EfficientNet B0		EfficientNet B1		EfficientNet B2		EfficientNet B3		EfficientNet B4		EfficientNet B5		EfficientNet B6		EfficientNet B7		EfficientNet B8	
	Accuracy	Loss	Accuracy	Loss	Accuracy	Loss	Accuracy	Loss	Accuracy	Loss	Accuracy	Loss	Accuracy	Loss	Accuracy	Loss	Accuracy	Loss
1	95	5	97	3	96	4	97	3	98	2	98	2	98	2	99	1	97	3
10	97	3	96	4	95	5	97	3	96	4	98	2	97	3	96	4	99	1
20	97	3	96	4	96	4	96	4	98	2	98	2	99	1	99	1	100	0
30	95	5	96	4	97	3	97	3	98	2	98	2	99	1	97	3	99	1
40	97	3	97	3	96	4	96	4	97	3	96	4	97	3	99	1	100	0
50	97	3	96	4	95	5	96	4	96	4	98	2	98	2	96	4	98	2
60	97	3	96	4	97	3	96	4	96	4	96	4	99	1	99	1	97	3
70	97	3	97	3	96	4	96	4	96	4	96	4	98	2	96	4	98	2
80	97	3	97	3	96	4	96	4	98	2	97	3	98	2	99	1	99	1
90	97	3	97	3	96	4	96	4	97	3	96	4	99	1	98	2	98	2
100	95	5	97	3	96	4	96	4	96	4	97	3	99	1	96	4	98	2

**Table 5:** Proposed method compared with the related studies.

Authors	Result
Emeršič et al. [3]	30
Tian and Mu [4]	69.33
Raveane et al. [5]	98
Zhang and Mu [6]	99.11
Kohlakala and Coetzer [7]	95.63
Tomczyk and Szczepaniak [8]	NA
Alshazly et al. [9]	22
Alkababji and Mohammed [10]	97.8
Jamil et al. [11]	97
Hansley et al. [12]	NA
Average of our work	97.07

## Data Availability

Datasets used to support the findings of the study are publicly available.

## References

[1] Abaza A., Ross A., Hebert C., Harrison M. A. F., Nixon M. S. (2013). A survey on ear biometrics. *ACM Computing Surveys*.

[2] Bhanu C. (2009). Ear Biometrics. *Advances in Intelligent Systems and Computing*.

[3] Emeršič Ž., Štepec D., Štruc V., Peer P. Training Convolutional Neural Networks with Limited Training Data for Ear Recognition in the Wild.

[4] Tian L., Mu Z. Ear recognition based on deep convolutional network.

[5] Raveane W., Galdamez P. L., Gonzalez Arrieta M. A. (2019). Ear detection and localization with convolutional neural networks in natural images and videos. *Processes*.

[6] Zhang Y., Mu Z. (2017). Ear detection under uncontrolled conditions with multiple scale faster region-based convolutional neural networks. *Symmetry*.

[7] Kohlakala A., Coetzer J. (2021). Ear-based biometric authentication through the detection of prominent contours. *SAIEE Africa Research Journal*.

[8] Tomczyk A., Szczepaniak P. S. (2019). Ear detection using convolutional neural network on graphs with filter rotation. *Sensors*.

[9] Alshazly H., Linse C., Barth E., Martinetz T. (2019). Handcrafted versus cnn features for ear recognition. *Symmetry*.

[10] Alkababji A. M., Mohammed O. H. (2021). Real time ear recognition using deep learning. *Telkomnika*.

[11] Jamil N., Almisreb A., Ariffin S. M. Z. S. Z., Md Din N., Hamzah R. (2018). Can Convolution Neural Network (Cnn) Triumph in Ear Recognition of Uniform Illumination Invariant?. *Indonesian Journal of Electrical Engineering and Computer Science*.

[12] Hansley E. E., Segundo M. P., Sarkar S. (2018). Employing fusion of learned and handcrafted features for unconstrained ear recognition. *IET Biometrics*.

[13] Wang Z.-q., Yan X.-d. Multi-scale feature extraction algorithm of ear image.

[14] Vu N.-S., Dee H. M., Caplier A. (2012). Face recognition using the poem descriptor. *Pattern Recognition*.

[15] Chowdhury D. P., Bakshi S., Guo G., Sa P. K. (2018). On applicability of tunable filter bank based feature for ear biometrics: a study from constrained to unconstrained. *Journal of Medical Systems*.

[16] Kumar A. (2007). Iit delhi ear database version 1.0. https://webold.iitd.ac.in/biometrics/Database_Ear.htm.

[17] Zhang Y., Mu Z.-C., Yuan L., Yu C., Qing L. (2017). USTB-Helloear: A Large Database of Ear Images Photographed Under Uncontrolled Conditions. *Image and Graphics*.

[18] Emeršič Ž., Štruc V., Peer P. (2017). Ear recognition: more than a survey. *Neurocomputing*.

[19] Gonzalez E., Alvarez L., Mazorra L. (2012). Ami Ear Database. http://ctim.ulpgc.es/research_works/ami_ear_database/.

[20] Frejlichowski D., Tyszkiewicz N. (2010). The West Pomeranian university of Technology Ear Database – a Tool for Testing Biometric Algorithms. *Image Analysis and Recognition*.

[21] Emeršič Ž., Štepec D., Štruc V. The unconstrained ear recognition challenge.

[22] Hoang V. T. (2019). Earvn1.0: a new large-scale ear images dataset in the wild. *Data in Brief*.

[23] Emeršič v., Peer P. Ear biometric database in the wild.

[24] Carreira-Perpinan M. A. (1995). Compression Neural Networks for Feature Extraction: Application to Human Recognition from Ear Images.

[25] Raposo R., Hoyle E., Peixinho A., ProenĂsa H. Ubear: A Dataset of Ear Images Captured On-The-Move in Uncontrolled Conditions.

[26] Prakash S., Jayaraman U., Gupta P. Connected component based technique for automatic ear detection.

[27] Alberink I., Ruifrok A. (2007). Performance of the fearid earprint identification system. *Forensic Science International*.

[28] Yan P., Bowyer K. Empirical evaluation of advanced ear biometrics.

[29] Phillips P., Wechsler H., Huang J., Rauss P. J. (1998). The feret database and evaluation procedure for face-recognition algorithms. *Image and Vision Computing*.

[30] Sim T., Baker S., Bsat M. (January 2001). The Cmu Pose, Illumination, and Expression (Pie) Database of Human Faces.

[31] Messer K., Matas J., Kittler J., Luettin J., Maitre G. Xm2vtsdb: the extended m2vts database.

[32] Abaza A. (2008). *High Performance Image Processing Techniques in Automated Identification Systems*.

[33] Oloko-Oba M., Viriri S. (2021). Ensemble of efficientnets for the diagnosis of tuberculosis. *Computational Intelligence and Neuroscience*.

